# Investigate Pathogenic Mechanism of TXNDC5 in Rheumatoid Arthritis

**DOI:** 10.1371/journal.pone.0053301

**Published:** 2013-01-09

**Authors:** Lin Wang, Yabing Zheng, Hengwei Xu, Xinfeng Yan, Xiaotian Chang

**Affiliations:** 1 Research Center for Medicinal Biotechnology, Shandong Academy of Medical Sciences, Jinan, Shandong, P. R. China; 2 Department of Pathology, Medical School of Shandong University, Jinan, Shandong, P. R. China; 3 Medical Research Center of Shandong Provincial Qianfoshan Hospital, Shandong University, Jinan, Shandong, P. R. China; 4 Department of Pharmacy, Shandong Tumor Hospital, Jinan, Shandong, P. R. China; University of Sydney, Australia

## Abstract

Hypoxia stimulates synovial hypoperfusion in rheumatoid arthritis (RA). TXNDC5 stimulates cellular proliferation in hypoxic conditions. We previously detected increased TXNDC5 expression in synovial tissues and blood from RA patients and demonstrated that the gene encoding TXNDC5 increased RA risk. The present study investigated the pathogenic roles of TXNDC5 in RA. Transgenic mice that over-expressed TXNDC5 (TXNDC5-Tg) were generated using C57BL/6J mice and treated with bovine collagen II to induce arthritis (CIA). Synovial fibroblasts from RA patients (RASFs) were cultured and incubated with TXNDC5-siRNA or CoCl_2_, a chemical that induces hypoxia. CIA was observed in 80% of the TXNDC5-Tg, but only 20% of the wild-type mice (WT) developed CIA. The clinical arthritis scores reached 5 in the TXNDC5-Tg, but this index only reached 2 in the control mice. CIA TXNDC5-Tg exhibited clear pannus proliferation and bone erosion in joint tissues. A significant increase in CD4 T cells was observed in the thymus and spleen of TXNDC5-Tg during CIA. Serum levels of anti-collagen II IgG, IgG1 and IgG2a antibodies were significantly elevated in the mice. Increased cell proliferation, cell migration and TXNDC5 expression were observed in RASFs following incubation with 1 µM CoCl_2_. However, this effect was diminished when TXNDC5 expression was inhibited with 100 nM siRNA. TNF-alpha, IL-1α, IL-1β and IL-17 levels were significantly increased in the blood of TXNDC5-Tg mice, but the levels of these cytokines declined in the supernatant of RASFs that were treated with TXNDC5 siRNA. The expression of adiponectin, a cytokine-like mediator, decreased significantly in RASFs following TXNDC5 siRNA treatment. These results suggest that TXNDC5-over-expressing mice were susceptible to CIA. This study also suggests that hypoxia induced TXCNDC5 expression, which contributed to adiponectin expression, cytokine production and the cellular proliferation and migration of fibroblasts in RA.

## Introduction

Thioredoxin domain-containing protein 5 (TXNDC5) is a protein-disulfide isomerase in the thioredoxin family. Members of this family interact with a broad range of proteins to reversibly oxidize two cysteine thiol groups to a disulfide bridge via a redox mechanism [1]. TXNDC5 expression is up-regulated by hypoxia and protects endothelial cells from hypoxia-induced cell death [2]. Rheumatoid arthritis (RA) decreases the oxygen supply in synovial tissues, which leads to synovial hypoxia and hypoperfusion [3]. We previously used proteomics to detect an increase in TXNDC5 expression in the synovial tissues of RA patients. We also detected significantly elevated TXNDC5 levels in the synovial fluids and blood of RA patients [4]. We observed that 9 SNPs in the TXNDC5 encoding gene exhibit a significant association with RA risk [5].

The present study used C57BL/6J mice to establish a transgenic line that over-expressed TXNDC5 (TXNDC5-Tg). These mice were injected with bovine collagen II to induce collagen-induced arthritis (CIA). C57BL/6J mice are generally resistant to CIA induced by bovine collagen [6]. The present study investigated whether TXNDC5 overexpression rendered C57 mice susceptible to CIA.

The present study suppressed TXNDC5 expression in primary cultured synovial fibroblasts from RA patients (RASFs) using RNA interference. We also treated RASF cells with CoCl2, which is a chemical inducer of hypoxia [7]. We examined the proliferative and invasive ability and cytokine production of these cultured cells in the presence of CoCl2 and TXNDC5 siRNA. This study investigated effect of TXNDC5 on RA in hypoxic conditions.

Charlton et al. observed that TXNDC5 interacts with the N-terminal residues of AdipoR1 using co-immunoprecipitation and mass spectrometry. The transient knockdown of TXNDC5 in HeLa cells increases AdipoR1 and AdipoR2 levels, which correlates with increased adiponectin-stimulated AMPK phosphorylation [8], [9]. Adiponectin is a cytokine-like mediator that is produced primarily in adipose tissue and synovial cells, and it stimulates the secretion of chemokines, proinflammatory cytokines, prostaglandin synthases, growth factors and factors related to bone metabolism and matrix remodeling from synovial fibroblasts in RA [10]. The present study investigated the pathogenic pathway of TXNDC5 in RA.

## Results

### Preparation of transgenic mice for the over-expression of TXNDC5

Four transgenic founders (numbers 5, 11, 14, and 15) carrying the TXNDC5 gene were identified using PCR with genomic DNA. The transgenic founder (F0) mice were mated with wild-type C57BL/6J mice to produce the F1 generation, and the TXNDC5 insert was detected in the F1 transgenic mice.

Synovial tissues of the F1 mice (n = 5) were dissected and subjected to real-time PCR to determine TXNDC5 expression. The expression of TXNDC5 was significantly increased in the synovial membranes of TXNDC5-Tg compared to the synovial samples from wild-type mice (WT), (p = 0.014) (**File S1A**). The translational level of TXNDC5 was also examined in the lung, kidney, spleen, thymus and liver of transgenic mice using Western blot analysis. High levels of TXNDC5 proteins (53 kDa) were observed in the spleen (p = 0.0081), thymus (p = 0.004) and kidney (p = 0.02) of TXNDC5-Tg (n = 5) compared to WT (n = 5). TXNDC5 expression was low in other organs of TXNDC5-Tg, such as the lung and liver, and no significant difference in the expression levels of this protein were observed between TXNDC5-Tg and WT mice (**File S1B**).

### Collagen-induced arthritis in TXNDC5-Tg mice

**Figure 1:**
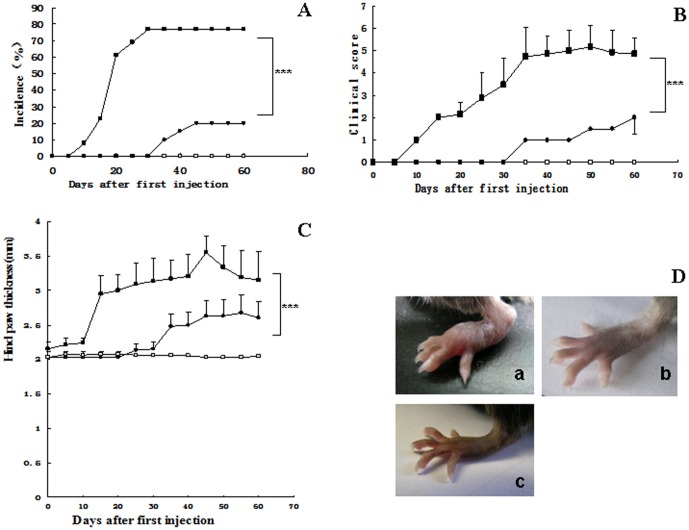
Clinical arthritis symptoms in collagen-injected TXNDC5-Tg mice. The incidence of arthritis (A), the hind paw clinical score (B) and the hind paw thickness (C) in TXNDC5-Tg (filled squares, n = 15) and WT (filled circles, n = 10) mice were measured at various time points following the first injection. A separate group of TXNDC5-Tg mice received BSA, and these mice were used as normal controls (empty squares, n = 10). (D) The symptoms of CIA were observed in the hind paws of these collagen-treated mice. a) represents collagen-injected TXNDC5-Tg, b) represents BSA-treated TXNDC5-Tg, c) represents collagen-injected wild type. * = p<0.05, ** = p<0.01, *** = p<0.001.

Obvious arthritis was observed in 2 of 15 TXNDC5-Tg mice 15 days after the first injection of bovine collagen II, and 80% (12/15) of these mice exhibited arthritis 30 days post-injection. CIA was observed in 1 of 10 WT 35 days after the first injection, and 20% (2/10) of WT exhibited CIA 45 days after injection. No obvious CIA was observed in BSA-treated TXNDC5-Tg (0%, 0/10) (**Figure 1A**). The clinical arthritis scores reached 5 35 days after the first injection in TXNDC5-Tg. However, this index was 1 in WT on the same day. The clinical score reached 2 in WT 50 days after the first injection (**Figure 1B**). The hind paw thickness was 3 mm in the TXNDC5-Tg 15 days after the first injection and reached 3.5 mm by the 40th day post-injection. The thickness reached approximately 2.5 mm in WT 50 days after the first injection (**Figure 1C**). The hind paws developed obvious edema and redness with a pronounced loss of function in 40% (6/15) of the TXNDC5-Tg; these effects were not observed in the control mice (**Figure 1D**). All experimental mice were included in the clinical scoring and paw thickness measurements.

**Figure 2:**
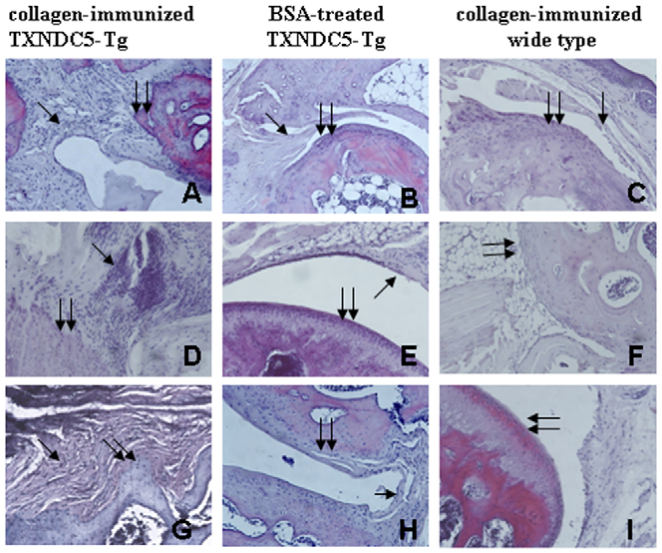
Histochemical examination of collagen-induced arthritis in experimental mice. The tissue structures of the paw (A–C), knee (E–F) and ankle (G–I) were histochemically examined. Synovial hyperplasia and inflammation, cartilage destruction and bone resorption with pannus formation were observed in the arthritic joints of the collagen-treated TXNDC5-Tg mice (n = 5) but not in WT (n = 5) and TXNDC5-Tg mice treated with BSA (n = 5). Single arrow represents synovial membrane, and double arrow indicates joint cartilage. Magnification: 200×.

Five mice were randomly selected from the collagen-injected TXNDC5-Tg, BSA-injected TXNDC5-Tg and WT groups to examine joint tissue structure. Histological examination revealed evident cartilage loss, synovial inflammation and bone destruction in all small joints of the hind paws of CIA TXNDC5-Tg. An abnormal proliferation of synovial fibroblasts, increased pannus and evident cellular invasion were also detected in the joints of the toe, knee and the ankle in these mice. Extensive pitting and ruffling were observed on the articular surfaces in the CIA TXNDC5-Tg mice. The joints of WT and BSA-injected TXNDC5-Tg exhibited normal smooth articular surfaces. No significant pannus or cellular infiltrates were observed in these controls. These results are presented in **Figure 2**. Similarly, 20% of WT exhibited mild joint inflammation following the collagen injection. Some cellular proliferation of synovial fibroblasts and T-cell invasion were observed, but no significant cartilage loss or bone destruction was observed in the joints of WT.

Immunohistochemistry detected TXNDC5 and adiponectin expression in the joint tissues of the transgenic mice. Intense TXNDC5 expression was observed in the synovial lining and the sub-lining cells of the joints in TXNDC5-Tg following collagen treatment (n = 5) (**File S2A**). TXNDC5 expression was very low in WT (n = 5) (**File S2B**) and BSA-treated TXNDC5-Tg mice (n = 5) (**File S2C**). Adiponectin expression was also detected in the synovial lining and the sub-lining cells of the joints of TXNDC5-Tg following collagen injection (**File S2D**). However, adiponectin expression was very low or undetectable in WT (**File S2E**), and BSA-treated TXNDC5-Tg mice (**File S2F**). No staining was observed when the primary antibodies were omitted or replaced by normal mouse serum.

**Figure 3:**
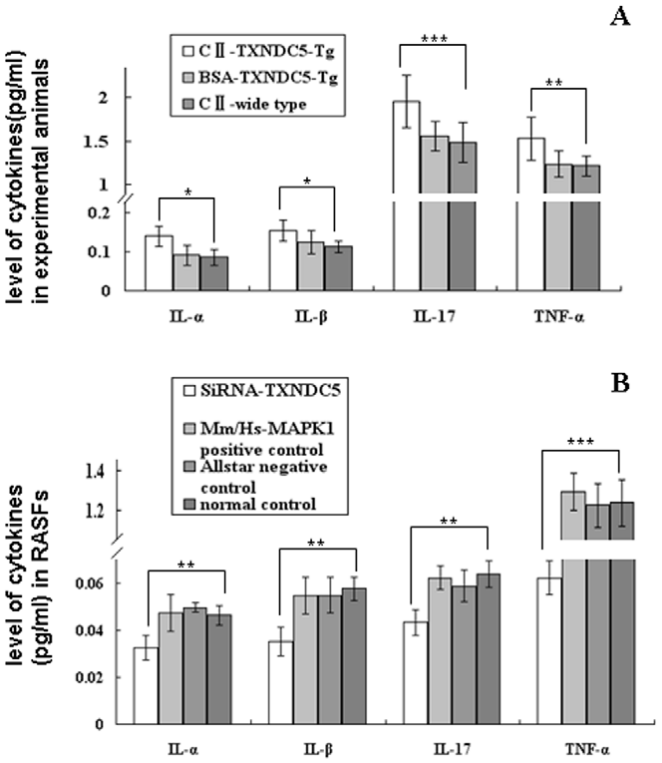
Measurement of cytokine levels using ELISA. (A) IL-1α, IL-1β, TNF-α and IL-17 levels in the sera of TXNDC5-Tg (n = 5) and WT mice following the injection of bovine collagen II (n = 5) and TXNDC5-Tg mice injected with BSA (n = 5). TXNDC5-Tg mice exhibited higher sera levels of TNF-α, IL-1α, IL-1β and IL-17 than wild type mice with treatment. (B) IL-1α, IL-1β, TNF-α and IL-17 levels in the supernatant of TXNDC5 siRNA (100 nM)-treated RASFs (n = 15). Parallel experiments with Mm/Hs-MAPK1 siRNA and Allstar siRNA were used as controls. RASFs without siRNA were also used as controls. TNF-α, IL-1α, IL-1β and IL-17 levels decreased significantly in RASF supernatants following treatment with TXNDC5 siRNA compared to the normal controls without the siRNA treatment. * = p<0.05, ** p<0.01, *** = p<0.001.

The cytokine levels in blood samples of collagen-treated TXNDC5-Tg (n = 5), collagen-treated WT (n = 5) and BSA-treated TXNDC5-Tg (n = 5) were investigated using ELISA. TXNDC5-Tg exhibited higher serum levels of TNF-α (p = 0.01), IL-1α (p = 0.02), IL-1β (p = 0.03) and IL-17 (p = 0.007) than WT. No significant differences in TNF-α, IL-1α, IL-1β and IL-17 levels were observed between collagen-treated WT and BSA-treated TXNDC5-Tg (p>0.05). These results are presented in **Figure 3A**.

The proportion of CD4 T cells in the TXNDC5-Tg CIA thymus was higher than in the control mice, including BSA-injected TXNDC5-Tg, BSA-injected WT and collagen-injected WT, but no significant changes in the proportion of CD8 T cells were observed between groups. The ratio of CD4/CD8 was notably increased. The proportion of CD4 T cells in the spleen of TXNDC5-Tg CIA mice was dramatically higher than control mice, but the number of CD8 T cells was not significantly different between groups. These results are presented in **File S3**.

The anti-collagen IgG, IgG1 and IgG2a levels were significantly increased in TXNDC5-Tg compared to control mice, including BSA-injected TXNDC5-Tg, BSA-injected WT and collagen-injected WT, 60 days after immunization (p<0.05). The increase in IgG2a levels in TXNDC5-Tg was most prominent after the immunization. In contrast, no significant differences in IgM levels were observed between TXNDC5-Tg and control mice. These results are presented in **File S4**.

### Suppression of TXNDC5 expression in RASFs using siRNA

**Figure 4:**
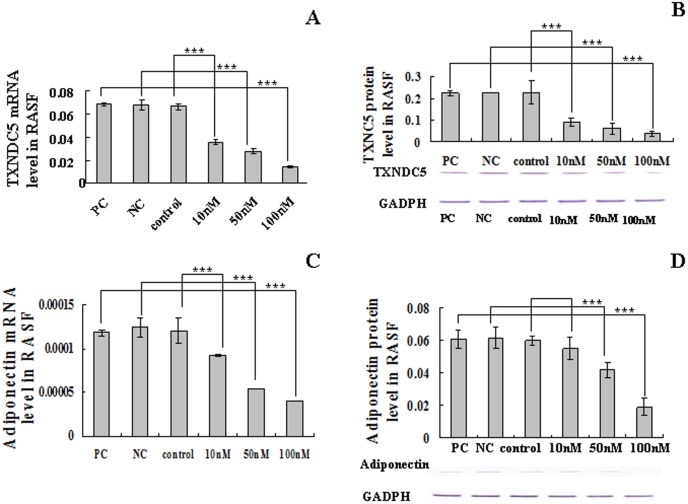
TXNDC5 and adiponectin expression in TXNDC5 siRNA-treated RASFs. The RASFs were transiently transfected with TXNDC5 siRNA (n = 15), and the effects of TXNDC5 knockdown were determined at the mRNA (A) and protein levels (B) using real-time PCR and Western blotting, respectively. The effect of inhibiting TXNDC5 expression on adiponectin expression was also determined using real-time PCR (C) and Western blotting (D). The mRNA levels of TXNDC5 and adiponectin were normalized to β-actin. The protein levels of TXNDC5 and adiponectin were normalized to GAPDH. TXNDC5 and adiponectin levels were not significantly changed between the positive control, the negative control and the cells without siRNA treatment. PC indicates the positive control, and NC indicates the negative control. * = p<0.05, ** p<0.01, *** = p<0.001.

The transfection of RASFs (n = 15) with siRNA at 10, 50 and 100 nM considerably reduced the expression of TXNDC5 protein and mRNA levels compared to the cells without siRNA treatment (p<0.05). siRNA treatment at 100 nM induced a strong inhibition of TXNDC5 mRNA (p = 0.00001) and protein expression (p = 0.006). These results are presented **Figures 4A and B**. The transcription of adiponectin decreased significantly in RASFs following the inhibition of TXNDC5 expression using 100 nM siRNA for 48 h (p = 0.00001). Decreased adiponectin protein expression was also detected in these cells (p = 0.002) (**Figures 4C, D**). TXNDC5 and adiponectin levels were not significantly altered between positive and negative control cells and the cells without siRNA treatment. The expression of MAPK1 significantly decreased following the transfection of Mm/Hs_MAPK1 control siRNA (results not shown), and AllStars negative control siRNA did not alter the gene expression. These results support the specificity of the RNA interference in these experiments.

TNF-α (p = 0.00001), IL-1α (p = 0.0034), IL-1β (p = 0.00001), and IL-17 (p = 0.00001) levels were significantly decreased in the supernatant of RASF cultures following treatment with 100 nM TXNDC5 siRNA compared to control RASFs without siRNA treatment. Parallel experiments with Mm/Hs-MAPK1 siRNA and Allstar siRNA were used as controls. No significant differences in TNF-α, IL-1α, IL-1β, and IL-17 levels were observed between these controls. These results are presented in **Figure 3B**.

### Hypoxia induction in CoCl2-treated RASFs

**Figure 5:**
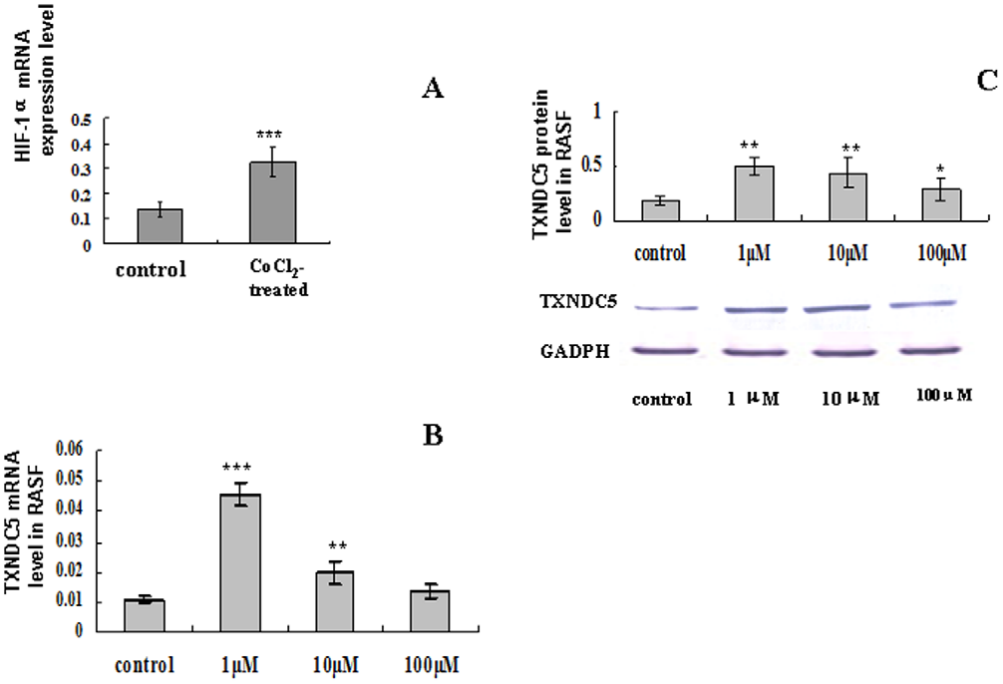
TXNDC5 expression in RASFs (n = 15) following treatment with different concentrations of CoCl2. (A) HIF-1α expression was detected in RASFs prior to and following 1 µM CoCl2 treatment. Real-time PCR and Western blotting analyses were used to measure TXNDC5 mRNA (B) and protein expression (C). * = p<0.05, ** p<0.01, *** = p<0.001.

Cultured RASFs cells (n = 15) were incubated with 1, 10 or 100 µM CoCl_2_ for 48 h. HIF-1α transcription increased significantly in CoCl_2_-treated RASFs cells compared to CoCl_2_ un-treated controls (p = 0.000126), which indicated the hypoxic state of treated cells (**Figure 5A**). TXNDC5 mRNA and protein levels increased significantly following CoCl_2_ stimulation (p<0.05) (**Figures 5B, C**). CoCl_2_ (1 µM) induced the highest TXNDC5 expression at transcriptional (p = 0.003) and translational (p = 0.01) levels.

### Cell proliferation, migration and RASF invasion in the presence of CoCl2 and TXNDC5 siRNA

**Figure 6:**
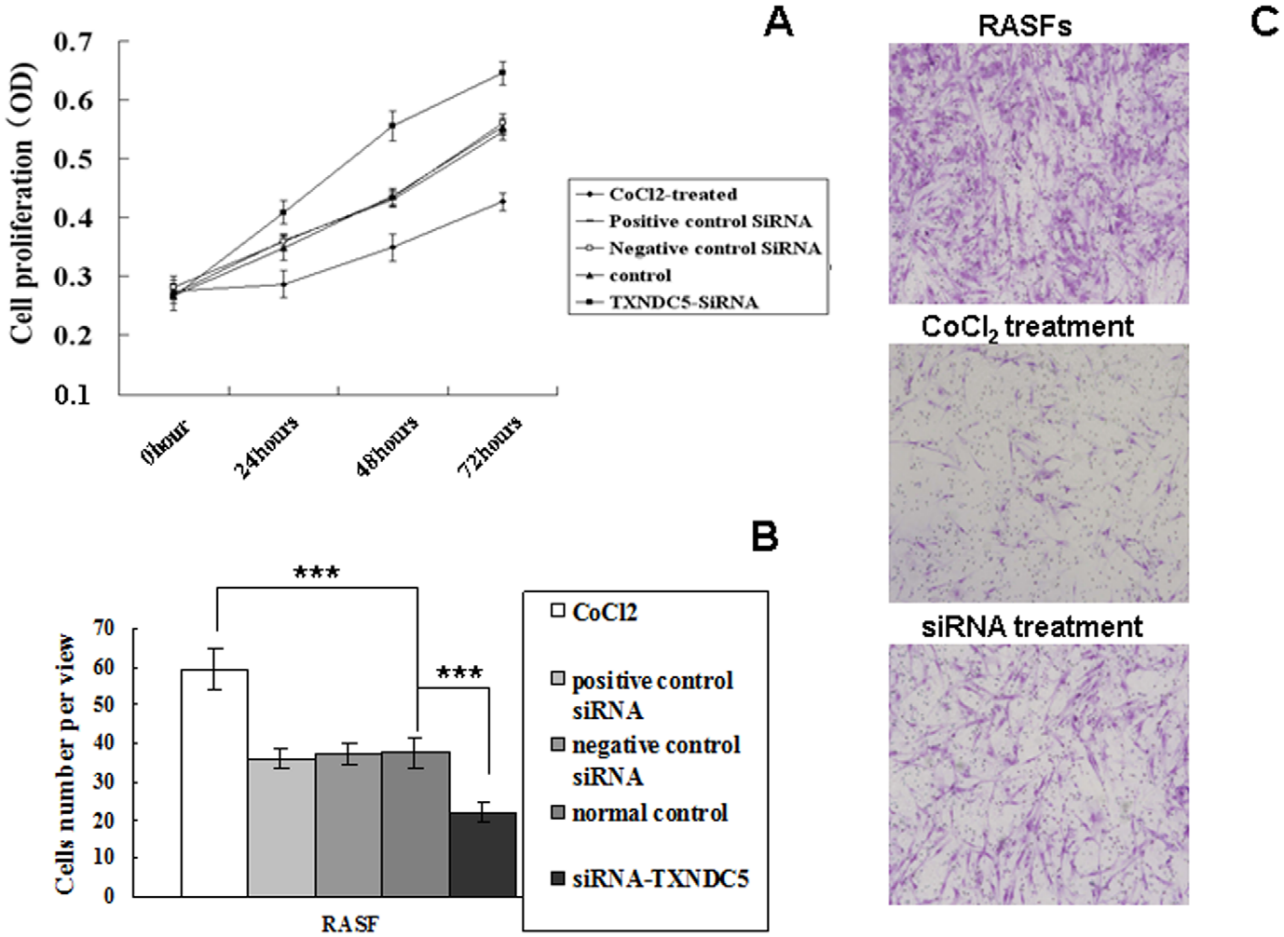
Cell proliferation and cell invasion in RASFs (n = 15) treated with CoCl2 and TXNDC5-siRNA. (A) The cell viability of RASFs was determined using an MTT assay. (B) The invasive ability of RASFs was examined using a 2-compartment transwell system. The average number of cells that invaded through the filter was quantified. (C) Crystal violet staining of the lower surface filters revealed the cells that passed through the filter and attached to the lower side of the filter (100×). The data were obtained from three independent experiments. * = p<0.05, ** p<0.01, *** = p<0.001.

A cellular proliferation assay was performed using cultured RASFs (n = 15) treated with TXNDC5 siRNA or CoCl_2_. RASFs incubated with 100 nM TXNDC5-siRNA exhibited a significant decrease in cell proliferation compared to the positive and negative controls (p = 0.005). Positive and negative siRNA controls exhibited no significant changes in cell proliferation. However, cellular proliferation increased significantly in the presence of 1 µM CoCl_2_ for 48 h compared to cells in the normal incubation condition (p = 0.002). These results are presented in **Figure 6A**.

The invasive ability of RASFs was examined using a 2-compartment transwell system. CoCl_2_ triggered a significant transmigration response in RASFs compared to basal transmigration (p = 0.001). In contrast, RASF migration decreased significantly when TXNDC5 expression was suppressed by TXNDC5 siRNA (p = 0.002). These results are presented in **Figures 6B and C** **.**

## Discussion

C57BL/6 TXNDC5-Tg and WT were subjected to CIA in the present study. CIA was observed in 80% of the TXNDC5-Tg, but only 20% of WT exhibited CIA. The clinical arthritis scores reached 5 in the TXNDC5-Tg, but this index only reached 2 in the control mice. The hind paws of the CIA TXNDC5-Tg mice developed evident edema and redness with a pronounced loss of function. Immunohistochemical analyses revealed that the paw, ankle and knee joints from CIA TXNDC5-Tg exhibited clear pannus proliferation, cell invasion and bone erosion, and these effects were not observed in WT. CIA in WT exhibited a lower severity of inflammation than TXNDC5-Tg. These results indicated that transgenic TXNDC5 over-expressing mice developed arthritis more rapidly and more severely than control mice, which suggests that TXNDC5 over-expression accelerated the onset of arthritis and increased disease incidence.

RASFs were treated with CoCl2 to mimic hypoxia in the present study. TXNDC5 expression was up-regulated in both RASFs Following this treatment. HIF-1 alpha levels also increased following CoCl_2_ treatment, which indicated the initiation of hypoxia. Greater cell proliferation and invasion were also observed in hypoxic cells. The growth and invasive potential of RASFs decreased following incubation with TXNDC5-siRNA. Our study also detected high TXNDC5 expression in the synovial tissues of TXNDC5-Tg. These results suggest that TXNDC5 promoted the proliferative and invasive abilities of synovial cells. Zhang et al. demonstrated that TXNDC5 exerted a strong effect on gastric cell proliferation, which enhanced the invasive capability of gastric cancer cells [11]. These data suggest that hypoxia induces TXNDC5 expression, and the inhibition of TXNDC5 expression prevents hypoxia-induced cell proliferation and migration.

Frommer et al. demonstrated the presence of adiponectin in the inflamed synovium at sites of cartilage invasion, lymphocyte infiltrates and perivascular areas. Adiponectin stimulates the secretion of chemokines, proinflammatory cytokines, prostaglandin synthases, growth factors and factors involved in bone metabolism and matrix remodeling from synovial fibroblasts [10]. Choi et al. also reported the contribution of adiponectin to synovitis and joint destruction in RA by stimulating the expression of vascular endothelial growth factor and matrix metalloproteinases-1 and -13 in fibroblast-like synoviocytes [12]. The present study observed a decrease in adiponectin expression in TXNDC5 siRNA-treated RASFs. Immunohistochemical analyses revealed an intense expression of TXNDC5 in the synovial lining and the sub-lining cells in CIA TXNDC5-Tg. These results suggest that TXNDC5 expression mediates adiponectin expression in the RA synovium, as hypothesized in our previous work [5].

Various cell types in the inflamed synovium express pro-inflammatory cytokines, including TNF-α, IL-1α, IL-1β and IL-17 [13]. The combination of IL-17 with IL-1 or TNF-α often produces synergistic or additive effects on osteoclastogenesis and osteoclast function, which underlie the abnormal bone erosion in RA [14], [15]. TXNDC5 inhibition in the present study decreased TNF-α, IL-1α, IL-1β and IL-17 levels. IL-1α, IL-β, TNF-α and IL-17 levels also increased significantly in the blood of TXNDC5-Tg mice compared to control mice. A high expression of TXNDC5 was observed in the joint tissues of the TXNDC5-Tg mice with CIA. These results suggest that TXNDC5 altered RA-related cytokine secretion.

The arthritis that is induced by immunization with bovine collagen is heavily dependent on MHC genetics. Male DBA/1 mice expressing the H-2q allele are susceptible to collagen induction [16]. C57BL/6J mice express the H-2b haplotype, and these mice are generally resistant to CIA induced by bovine collagen [17], [18]. These results are consistent with the present study; the control WT exhibited an arthritis incidence of only 20%, and the generated transgenic over-expressing TXNDC5 mice exhibited an arthritis incidence of 80%. These results suggest that TXNDC5 over-expression rendered the mice susceptible to disease. TXNDC5 overexpression may overcome the H-2q allele to induce arthritis in C57 mice.

CD4 T cells differentiate into distinct phenotypes that secrete characteristic patterns of cytokines. These cytokines promote immune responses by aiding the activation and maintenance of CD8 T cells and macrophages and mediating B cell antibody production and class switching [19], [20]. T cells contribute to synovitis and joint destruction in RA [19], [21]. Activated CD4 T cells produce interleukin-2 and other inflammatory cytokines, which initially stimulate monocytes, macrophages and synovial fibroblasts. Subsequently, activated macrophages and synovial fibroblasts induce the overproduction of inflammatory cytokines, such as TNF-α, IL-1β, IL-6, IL-15 and IL-18 [22]–[25]. A significantly higher frequency of thymic and splenic CD4 T cells was detected in TXNDC5-Tg in the present study, but CD8 cells were less influenced than CD4 cells in each group. CD4 T cells may be required for the induction of CIA, and CD8 T cells may play a suppressive role in the etiology of CIA [26]. CD4 T cells contribute to autoantibody production, inflammation, synovial angiogenesis, hyperplasia and cartilage and bone destruction in CIA and RA [27]. RA patients exhibit significantly reduced numbers of CD8 T cells and greater CD4/CD8 ratios in peripheral blood and a greater number of CD4 T cells and lower CD4/CD8 ratios in synovial fluid [28]–[32].

RA is characterized by persistent synovitis, systemic inflammation, and autoantibodies particularly to collagen and citrullinated peptides. The present study measured the generation of collagen-specific IgG antibodies in TXNDC5-Tg after primary immunization. The anti-collagen IgG, IgG1 and IgG2a levels were significantly increased in TXNDC5-Tg, which reflected greater T-cell activation in TXNDC5-Tg following booster immunizations. The production of anti-collagen antibodies is a major factor in the susceptibility to CIA. High levels of circulating anti-collagen antibodies invariably accompany the development of CIA and may be required for the development of RA [33], [34].

In conclusion, TXNDC5-Tg mice exhibited an enhanced susceptibility and severity of CIA. The present study also suggested that hypoxia induced TXCNDC5 expression, which contributed to adiponectin expression, cytokine production and the cellular proliferation and migration of fibroblasts derived from RA patients. These results improve our understanding of the pathogenic mechanisms of RA.

## Materials and Methods

### Generation of TXNDC5 over-expressing transgenic mice

Full-length mouse TXNDC5 cDNA was cloned from neonate C57BL/6J mouse tissue using an RT-PCR kit from Invitrogen (USA), and the cDNA clones were confirmed using sequencing analysis. The TXNDC5 open reading frame (ORF) was linked into the pCDNA3.1(+) plasmid (Invitrogen) using PCR. The TXNDC5 cDNA was inserted downstream of the cytomegalovirus (CMV) immediate-early promoter in this vector between the BamHI and XhoI cloning sites. The completed plasmid was linearized using Endonuclease BglII//PvuII digestion to remove the prokaryotic sequence. The DNA fragments were gel-purified, dissolved in Tris-HCl EDTA at a final concentration of 5 ng/µl, and injected into the pronucleus of fertilized zygotes harvested from C57BL/6J mice that were obtained by the crossing of C57Bl/6 (female) mice with dBa/2 (male) mice. The injected egg was transferred into the oviducts of a pseudo-pregnant recipient female. This procedure is the conventional method for the generation of transgenic mice. This experiment was completed in Dr. Zhang Lianfeng's laboratory at The Institute of Laboratory Animal Science, Chinese Academy of Medical Sciences.

The transgenic founder (F0) mice were mated with wild-type C57BL/6J mice to produce the F1 generation. Mouse genomic DNA was first extracted from tail biopsies. The TXNDC5 insert was examined in F1 transgenic mice using PCR with the following primers: sense, 5′-GCAAGCTTCATGAGACCTAGAATGAAGTAT-3′; antisense, 5′-CGGGATCCTTTACTGCATTAGATTTTCGAG-3′. The PCR product was confirmed by sequencing analysis. The F1 transgenic lines were mated with wild-type C57BL/6J mice to produce F2 mice. The colony was maintained by breeding the transgenic animals to wild type (C57BJ/6) mice. The mice were housed at an Institutional Animal Care and Use Committee (IACUC), and the Ethics Committee of Shandong Provincial Qianfoshan Hospital approved the study.

### Collagen-induced arthritis in transgenic animals

TXNDC5-Tg (n = 15) and WT (n = 10) on the C57BL/6 background (8–12 weeks old, male) were injected intradermally at the base of the tail with 200 µl of 2 mg/ml bovine type II collagen, which was dissolved in 10 mM acetic acid (4 mg/ml overnight at 4°C) and emulsified in complete Freund's adjuvant containing 1 mg/ml heat-inactivated mycobacterium tuberculosis (Sigma-Aldrich, USA). The mice were immunized with bovine type II collagen in incomplete Freund's adjuvant in the same manner 21 days later. The thickness of the hind paws was measured using Vernier calipers. The clinical arthritis score was calculated as described previously [35]. Each paw score was based on the degree of swelling and periarticular erythema using a scale of 0–3 as follows: 0 = no evidence of erythema or swelling, 1 = erythema confined to one joint region only, 2 = erythema and swelling limited to one joint region only, and 3 = severe erythema and swelling extending from the ankle to the midfoot (tarsal) joint involving both joint regions. Scores from all four paws were added to provide a total score for each mouse. The maximum possible score per mouse was 12. The mice were sacrificed 60 days after the first collagen injection, and their joints were dissected, decalcified, sectioned and stained with hematoxylin and eosin using established protocols. Control joints were obtained from untreated wild type and immunized wild type mice that did not exhibit clinical signs of arthritis. A separate group of TXNDC5-Tg mice (n = 10) were treated with BSA as controls.

The Ethics Committee of Shandong Provincial Qianfoshan Hospital approved the study protocol.

### Human tissue samples

Synovial tissue samples were collected during knee joint replacement surgery from patients with RA (n = 15, 9 female, 23–68 yrs old, mean 49 yrs). All of the patients fulfilled the ACR (American College of Rheumatology) diagnosis criteria for RA. The RA patients exhibited a disease duration of three to nine years and were classified as erosive RA (Larsen class IV–V). These patients presented high levels of C-reactive protein (12–320 mg/L, mean 59 mg/L), anti-CCP (16–470 U/ml, mean 290.4 U/ml) and RF (40–320 U/ml, mean 171.2 U/ml). All of the patients provided written informed consent to participate in this study. The Ethics Committee of Shandong Provincial Qianfoshan Hospital approved this study.

### Culture of synovial fibroblasts

Synovial tissues were finely minced and incubated with a solution containing 1 mg/ml collagenase (type II) (Sigma-Aldrich) in Dulbecco's modified Eagle's medium (DMEM, Invitrogen, USA) for 3 h in a 37°C, 5% CO2 incubator (Thermo, USA). An equal volume of PBS solution containing 0.25% trypsin (Solabio, China) was added to the culture. These cells were cultured overnight, and the non-adherent cells were floated and removed.

### Inhibition of TXNDC5 expression with siRNAs

siRNA oligonucleotides targeting TXNDC5 (target mRNA sequence: 5′-CACATACAGGCTTAAGCTCTA-3′) were designed and synthesized by QIAGEN (Germany). Cultured RASFs were transfected with siRNAs at 10, 50 and 100 nM using a HiPerFect transfection reagent (QIAGEN) according to the manufacturer's protocol. The cells were harvested for analysis 48 h following the transfection. Parallel experiments with Mm/Hs-MAPK1 siRNA (AATGCTGACTCCAAAGCTCTG) and Allstar siRNA were provided with the kit, and these sequences were used as positive and negative controls, respectively. The control RNAs were used at the same concentrations as TXNDC5 siRNA.

### Hypoxia induction in cultured RASFs

RASFs (n = 15) were incubated in the presence of cobalt chloride (CoCl_2_) at concentrations of 1, 10 or 100 µM (Sigma, USA) for 48 h under normal conditions.

### Cell proliferation assay

The RASFs were seeded onto 96-well culture plates and incubated until they reached 80% confluence. The cultures were treated with TXNDC5 siRNA (100 nM) or CoCl_2_ (1 µM). The cells were harvested at 48 h and subjected to an MTT [3-(4,5-dimethylthiazol-2-yl)-2,5-diphenyltetrazolium bromide] assay. A total of 100 µl of 1 mg/ml MTT (Amoresco, USA) was constituted in the culture medium, added to each well and incubated for 4 h at 37°C in the dark. The MTT solution was removed, and the cells were washed twice in PBS followed by air-drying. The MTT-formazan products were extracted with 100 µl DMSO in the dark at room temperature. The absorbance was measured at 490 nm using a spectrophotometer.

### Cell invasion assay

The cell invasion assay was performed in a transwell apparatus (Costar Corning, USA). RASFs at a density of 3×104 cells/well were grown to confluence and incubated with 100 nM of siRNA or 1 µM CoCl_2_ in the upper compartment of the transwell apparatus for 48 h. The lower compartment was filled with DMEM with 10% FBS, and the plates were continually incubated at 37°C for 24 h. The upper surface of the insert was wiped with cotton swabs to remove non-invading cells, and the bottom surface of the insert was stained with Giemsa. The number of cells that invaded through the membrane was quantified in 5 random fields at 100× magnification.

### Western blotting

The cultured cells and animal tissues were homogenized in RIPA buffer (Beyotime, China) supplemented with protease and phosphatase inhibitors on ice and centrifuged at 16000× g for 5 min at 4°C. Thirty micrograms of the total protein was separated using sodium dodecyl sulfate-polyacrylamide gel electrophoresis (SDS-PAGE) and trans-blotted onto nitrocellulose membranes (Amersham, USA). Western blot analyses were conducted using antibodies against human TXNDC5 at a 2,000-fold dilution or adiponectin at a dilution of 1∶1000 overnight at 4°C. The anti-TXNDC5 antibody (Abcam, USA) was raised in goats using an oligopeptide (SLHRFVLSQAKDEL) against TXNDC5, and the adiponectin antibody (Abcam) was prepared by immunizing a rabbit with a 15 amino acid peptide near the amino terminus. The immunosignals were visualized with the Protein Detector BCIP/NBT Western Blot Kit (Beyotime) following the manufacturer's instructions. A separate membrane was prepared using the same protocol and probed with an anti-GADPH antibody (Santa Cruz, USA) to normalize sample loading.

### Immunohistochemistry

Whole ankle joints were collected from sacrificed animals and fixed, decalcified, embedded in paraffin, sectioned and subjected to histopathological examination. The tissue sections were heated at 95°C for 10 min in a citrate buffer solution (Sigma) for antigen recovery and subsequently incubated with an endogenous peroxidase inhibitor (MaixinBio, China) for 30 min at room temperature. The sections were washed with PBS buffer (0.132 M NaCl, 0.0066 M K2HPO_4_, and 0.0015 M KH2PO_4_ in distilled water, pH 7.6) and incubated with the anti-TXNDC5 and anti-adiponectin antibodies overnight at 4°C. The immunoreactions were processed using the UltraSensitive TM S-P Kit (Maixin-Bio, China) according to the manufacturer's instructions. The immunoreactive signals were visualized using a DAB substrate, which stains the target protein yellow. The cell structures were counterstained with hematoxylin. The tissue samples were incubated with goat pre-immune serum (Maixin-Bio) or treated with the modification buffer without the addition of the antibody to determine antibody specificity and optimize the antibody dilution.

### Real-time PCR

Total RNA was extracted from the cultured cells and animal tissues and reverse-transcribed using an RNA PCR Kit (TaKaRa, Japan). Real-time PCR was conducted using the LightCycler 480 (Roche, Germany). The relative mRNA expression was calculated using the comparative threshold cycle (Ct) method. The relative target gene expression was normalized relative to β-actin mRNA levels. The primer sequences for the amplification of TXNDC5 were as follows: forward primer 5′-CTCTGGGCCTTGAACATT-3′, and reverse primer 5′-CCCTCAGTGACTCCAAA-3′. The primer sequences for the amplification of human adiponectin were as followings: forward primer 5′-TATTGGTCCTAAGGGAGACATC-3′, and reverse primer 5′-TAAAGCGAATGGGCATGTT-3′. The primer sequences for the amplification of β-actin were as follows: forward primer 5′-TGGCACCCAGCACAATGAA-3′, and reverse primer 5′-CTAAGTCATAGTCC GCCTAGAAGCA-3′. The primer sequences for the amplification of hypoxia inducible factor-1α (HIF-1α were as follows: 5′-CAAAGTTGAATCAGAAGATACAAGTAG-3′, and reverse primer 5′-CTTCCTCAAGTTGCTGGTC-3′.

### Measurement of TNF-α, IL-1α, IL-β and IL-17 levels using enzyme-linked immunosorbent assay (ELISA)

RASFs were treated for 48 h with 100 nM siRNA, and the culture medium was collected and centrifuged at 6000 rpm for 10 min at 4°C. The serum samples were collected from the experimental animals. TNF-α, IL-1α, IL-β and IL-17 levels were measured using ELISA kits provided by R&D (USA).

### Measurement of serum anti-collagen antibody levels

CIA was induced in TXNDC5-Tg mice (n = 10) as described above. BSA-treated TXNDC5-Tg, collagen-treated WT and BSA-treated WT (n = 10 per group) mice were used as controls. Serum samples were collected from these mice prior to immunization and 60 days after primary immunization. Serum anti-collagen IgG, IgG1, IgG2a and IgM levels were measured as follows. ELISA plates (Dynex Technologies, Chantilly, VA) were coated overnight at 4°C with 10 mg/ml native bovine collagen in PBS. The plates were washed with PBS containing 0.05% Tween-20 (PBST), and nonspecific binding was blocked with PBS containing 2% skimmed milk for 1 h at room temperature. The plates were washed three times, and serum samples in a 50-fold dilution were added and incubated for 2 h at room temperature. The plates were washed three times, and HRP-conjugated goat anti-mouse total IgG (Sigma), HRP-conjugated goat anti-mouse total IgM (Sigma), HRP-conjugated goat anti-Mouse IgG1 (Southern Biotechnologies, Birmingham, AL) and HRP-conjugated goat anti-Mouse IgG 2a (Southern Biotechnologies, Birmingham, AL) in a 1000-fold dilution were added and incubated at room temperature for 1 h. The plates were washed three times, and developed using tetramethyl benzidine (TMB, Sigma) as a substrate. The OD was measured using a microplate reader.

### Fluorescence-activated cell sorting (FACS) analysis

CIA was induced in TXNDC5-Tg mice (n = 10) as described above. BSA-treated TXNDC5-Tg, collagen-treated WT and BSA-treated WT (n = 10 per group) mice were used as controls. The thymus and spleen were removed under sterile conditions, and lymphocytes were collected. The cells were suspended in a DMEM medium at a concentration of 1×107 cells/L. The cell suspension was purified using a lymphocyte-separating medium. The cells were washed with the PBS buffer three times. A 50-µl aliquot of lymphocyte suspension was transferred into a 12×75 mm polystyrene round-bottom tube, and 10 µl of antibody combinations were added to each tube. The sample was mixed gently, incubated for 20 min at 4°C, and analyzed using a flow cytometer (BD FACSAriaTM II). The following antibodies were used for FACS analysis: FITC Anti-Mouse CD4 (BD Pharmingen) and PerCP Anti-Mouse CD8a (BD Pharmingen).

### Statistical analyses

Statistical analyses of the data were performed using SPSS V.16 software (SPSS, USA). Multiple comparisons were conducted using ANOVA. T-tests assessed statistical differences between two groups. P values less than 0.05 were considered significant. The data are presented as standard deviations.

## Supporting Information

File S1 **TXNDC5 expression in various tissues of TXNDC5-Tg mice.** (A) The TXNDC5 transcriptional level was determined using real-time PCR in synovial tissues from TXNDC5-Tg (n = 5) and WT (n = 5) mice. (B) The translational level of TXNDC5 was examined using Western blotting of tissues from the spleen, thymus, kidney, liver and lung of TXNDC5-Tg (n = 5) and WT (n = 5) mice. * = p<0.05, ** = p<0.01, *** = p<0.001.
(TIF)

File S2 **Immunohistochemical detection of TXNDC5 and adiponectin in the knee joint tissues of experimental mice.** Tissue sections of knee joints were obtained from collagen-treated TXNDC5-Tg (A, D), wild type (B, E) and BSA-treated TXNDC5-Tg (C, F) mice. Sections A, B and C illustrate the results of TXNDC5 immunostaining, and sections D, E and F represent adiponectin immunostaining. Single arrow indicates synovial tissue, and double arrows indicate cartilage and bone. Magnification 200×.
(TIF)

File S3 **CD4 and CD8 T cell proportions in CIA mice.** The subsets of T cells in thymus (A, B) and spleen (C, D) of experimental animals (n = 10 per group) were detected using a flow cytometer. The percentage of CD4 T cells was significantly increased in CIA thymus and CIA spleen of TXNDC5-Tg mice, but none of the groups exhibited a significantly altered CD8 T cell proportion. Data are presented as the means±SD. * = p<0.05, ** = p<0.01.
(TIF)

File S4 **Collagen II-specific IgG and IgM responses in CIA mice.** Serum samples (n = 10 per group) were collected 60 days after immunization with collagen II. Anti-collagen IgG, IgG1, IgG2a and IgM levels were measured using ELISA. Anti-collagen IgG, IgG1 and IgG2a were significantly elevated in TXNDC5-Tg. Data are presented as the means±SD. * = p<0.05, ** = p<0.01.
(TIF)
