# Supplementary figures and images for: Investigate Pathogenic Mechanism of TXNDC5 in Rheumatoid Arthritis
Source: PLoS One. 2013 Jan 9;8(1):e53301. doi: 10.1371/journal.pone.0053301 (PMC3541148; doi:10.1371/journal.pone.0053301)

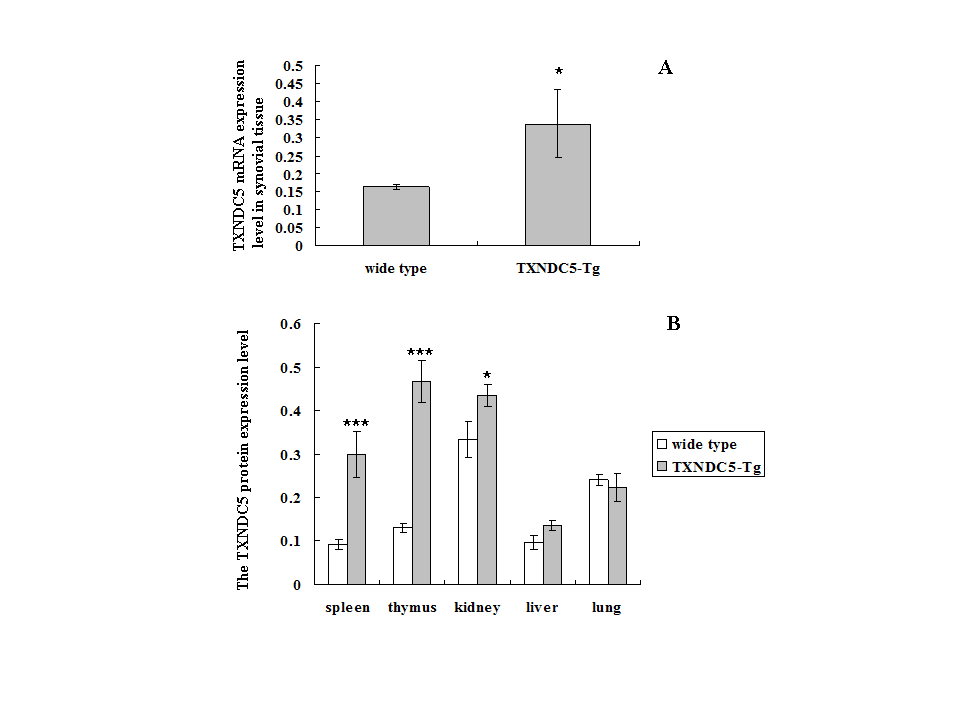

Supplement: File S1 — TXNDC5 expression in various tissues of TXNDC5-Tg mice. (A) The TXNDC5 transcriptional level was determined using real-time PCR in synovial tissues from TXNDC5-Tg (n = 5) and WT (n = 5) mice. (B) The translational level of TXNDC5 was examined using Western blotting of tissues from the spleen, thymus, kidney, liver and lung of TXNDC5-Tg (n = 5) and WT (n = 5) mice. * = p<0.05, ** = p<0.01, *** = p<0.001. (TIF) [file pone.0053301.s001.tif]

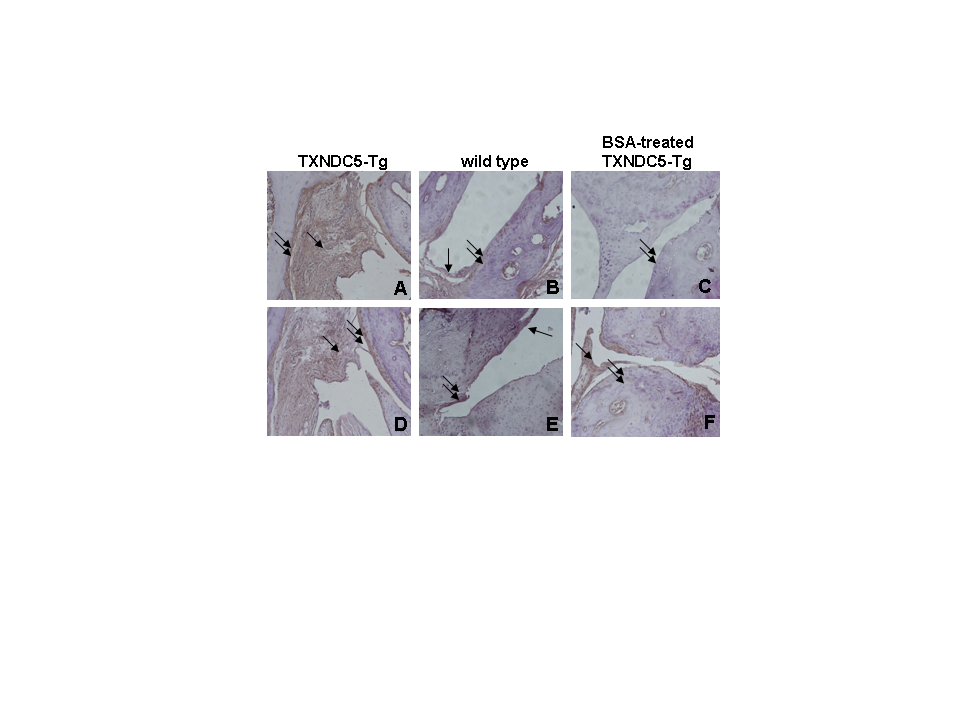

Supplement: File S2 — Immunohistochemical detection of TXNDC5 and adiponectin in the knee joint tissues of experimental mice. Tissue sections of knee joints were obtained from collagen-treated TXNDC5-Tg (A, D), wild type (B, E) and BSA-treated TXNDC5-Tg (C, F) mice. Sections A, B and C illustrate the results of TXNDC5 immunostaining, and sections D, E and F represent adiponectin immunostaining. Single arrow indicates synovial tissue, and double arrows indicate cartilage and bone. Magnification 200×. (TIF) [file pone.0053301.s002.tif]

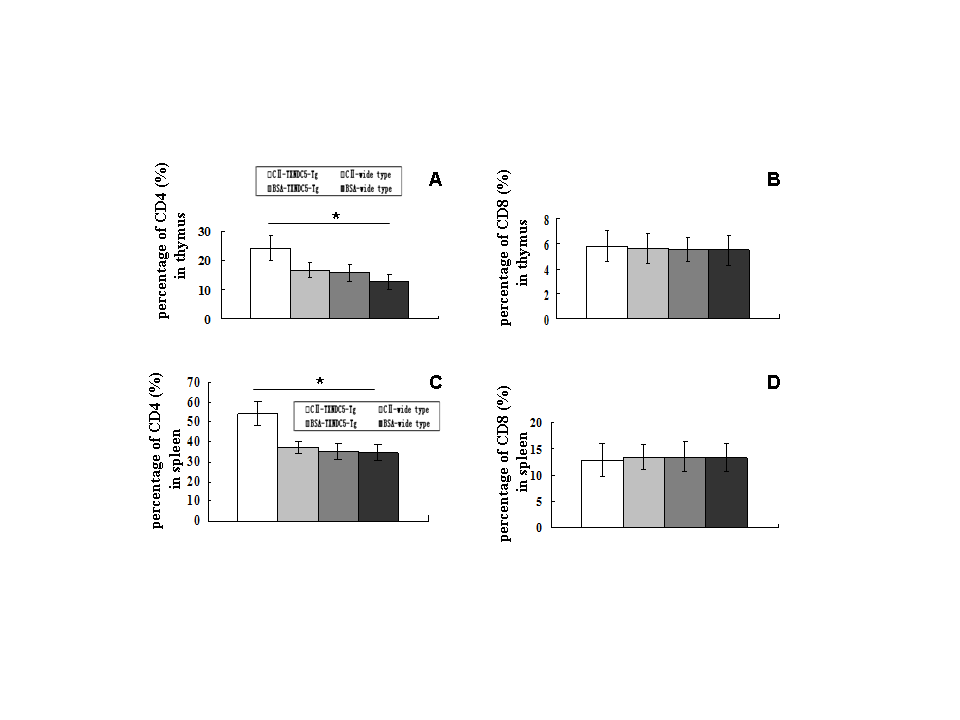

Supplement: File S3 — CD4 and CD8 T cell proportions in CIA mice. The subsets of T cells in thymus (A, B) and spleen (C, D) of experimental animals (n = 10 per group) were detected using a flow cytometer. The percentage of CD4 T cells was significantly increased in CIA thymus and CIA spleen of TXNDC5-Tg mice, but none of the groups exhibited a significantly altered CD8 T cell proportion. Data are presented as the means±SD. * = p<0.05, ** = p<0.01. (TIF) [file pone.0053301.s003.tif]

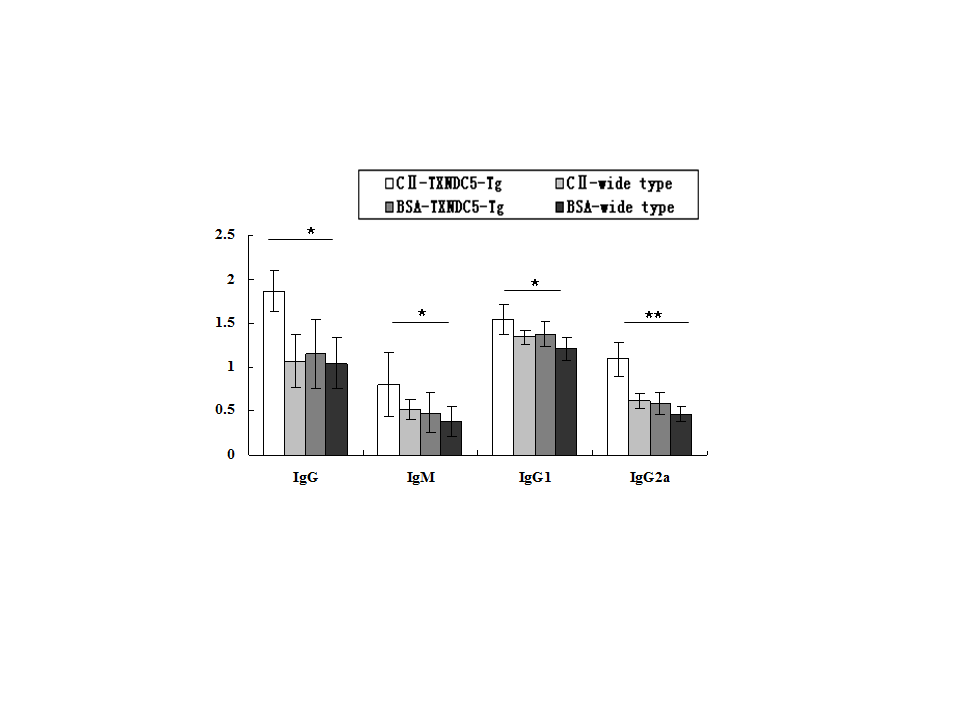

Supplement: File S4 — Collagen II-specific IgG and IgM responses in CIA mice. Serum samples (n = 10 per group) were collected 60 days after immunization with collagen II. Anti-collagen IgG, IgG1, IgG2a and IgM levels were measured using ELISA. Anti-collagen IgG, IgG1 and IgG2a were significantly elevated in TXNDC5-Tg. Data are presented as the means±SD. * = p<0.05, ** = p<0.01. (TIF) [file pone.0053301.s004.tif]
